# When “Myeloma” is not a Myeloma: a case report of malignant bone lymphoma

**DOI:** 10.1097/j.pbj.0000000000000261

**Published:** 2024-07-24

**Authors:** José Guilherme Freitas, Teresa Ribeiro, Cláudia Moreira, Ilídia Moreira, José Mário Mariz

**Affiliations:** a Onco-Hematology Department, Instituto Português de Oncologia do Porto FG, Porto, Portugal; b Hematology Department, Hospital Braga, Braga, Portugal

Malignant bone lymphoma (MBL) is a rare entity and is subdivided into primary (PBL) and secondary (SBL) subtypes.^1,2^ PBL accounts for 5% of MBL and presents as unifocal lesion (uPBL) with or without lymph node involvement or as multifocal disease (mPBL), also known as “multifocal osteolymphoma” or “polyostotic lymphoma.”^3,4^ SBL, defined by disseminated lymphoma with bone involvement, is much more common than PBL, accounting for 15% of cases.^2,5,6^ The median age at diagnosis varies between 40 and 60 years with a slight male predominance, diffuse large B-cell lymphoma (DLBCL) being the most common histological subtype (70–80% of cases).^3,7^

Patients may present with bone pain or tumor mass, the long bones, such as the femur and humerus, being the most commonly affected, whereas pathological fracture is the most frequent complication at diagnosis.^1,7–9^ Patients with PBL are staged according to the Ann Arbor system, although the *International Extranodal Lymphoma Study Group* (IELSG) developed an adapted staging system. PBL presenting as uPBL is classified as stage-IE in Ann Arbor and IELSG systems and mPBL as stage IV in Ann Arbor and IVE in IELSG system.^6^

Primary bone DLBCL should be treated with an anthracycline-containing chemotherapy, rituximab plus cyclophosphamide, doxorubicin, vincristine and prednisone (R-CHOP) with radiotherapy being the standard treatment approach.^10,11^ The overall response rate is over 90% and 5-year overall survival 80–90%,^7,12,13^ over 70–80% being for localized disease, less than 40% for stage IV, and even less for SBL.^6^ Older patients, pathologic bone fracture as the initial presentation, and a high *International Prognostic Index* (IPI) score portray poorer outcomes,^12^ although the role of IPI score as prognostic factor is controversial.^12,14^ Another issue to be considered is the role of bisphosphonates as their use on PBL is still not established.^15,16^

Most of the literature is exclusively based on small retrospective series and case reports, so biological, clinical, and therapeutic questions remain open to discussion.^3^ We describe a case of a patient presented with bone fractures and lytic lesions resembling multiple myeloma (MM).

A 63-year-old woman presented with a medical history of cirrhosis, diabetes mellitus, fibromyalgia, and acute myeloblastic leukemia (AML), French-American-British classification M2, diagnosed at 2000 and treated with a standard “7+3” regimen plus consolidation with high-dose cytarabine. Afterward, she had secondary cytopenias (neutropenia and thrombocytopenia) due to previous AML treatment, portal hypertension, and cirrhosis.

**Figure 1.**
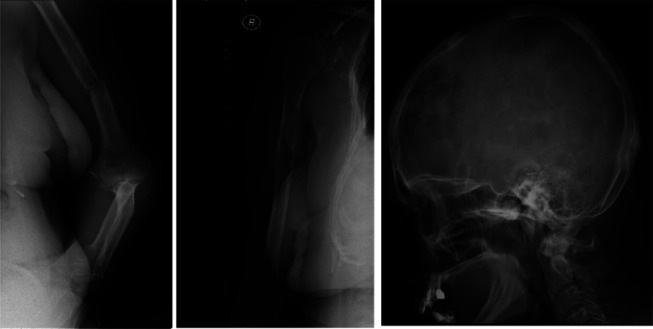
Skeleton X-ray: pathologic bone fracture of the left humerus (left one); multiple osteolytic lesions (middle: right humerus; right: skull).

**Figure 2.**
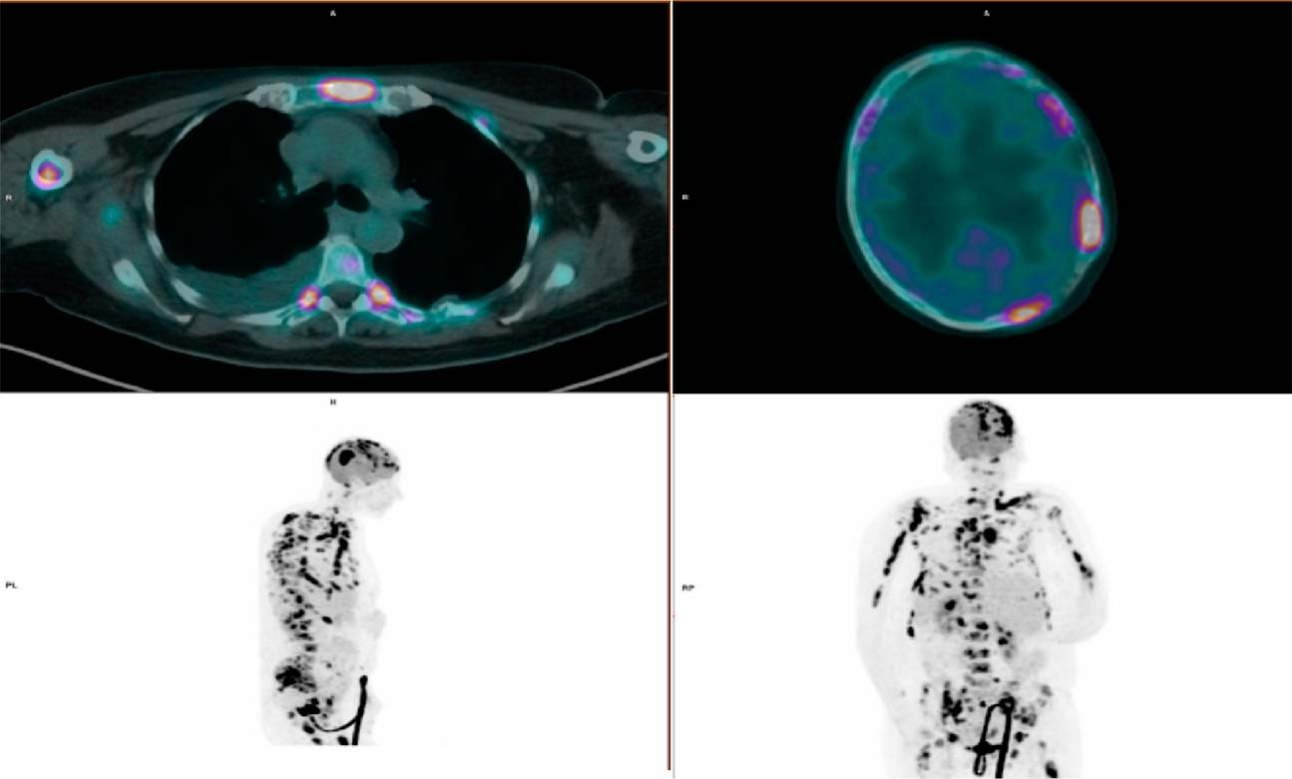
PET-CT scan: multiple osteolytic bone lesions with high avidity for 18F-FDG and low metabolism in the left cervical lymph node and right lower lobe.

The patient presented in our emergency department with generalized pain, fatigue, anorexia, and weight loss and a pathologic bone fracture in the right clavicle and left humerus in the previous month (Fig. 1). The physical examination did not reveal any palpable hepatosplenomegaly or lymphadenopathy. Blood tests showed a hemoglobin of 10.6 g/dL, white blood cell count 1.74 × 10^9^/L (neutrophil 0.82 × 10^9^/L; lymphocyte 0.31 × 10^9^/L), and platelet count (93 × 10^9^/L). Biochemistry did not reveal any hepatic or renal dysfunction; albumin was 3.2 g/dL [VR 3.8–5.3], lactate dehydrogenase 472 U/L [RV 67–248], and no hypercalcemia, hyperuricemia, or beta-2 microglobulin elevation. Serum quantitative immunoglobulins, serum protein, and 24-hour urine immunoelectrophoresis were normal. Skeleton X-ray showed osteolytic lesions on the right humerus and skull and bone fracture of the left humerus (Fig. 1), and a spine CT showed osteolytic lesions and compressive fractures at D7-D8. A PET-CT scan showed bone lesions with high avidity for 18F-FDG and low metabolism in the left cervical lymph node and right lower lobe (Fig. 2). Bone marrow aspirate and biopsy did not reveal plasma cell or lymphoid disorder. A bone biopsy of the manubrium revealed a diffuse infiltration of medium/large-sized lymphoid cells (CD10^+^, CD20^+^, BCL2+, BCL6+, MUM1+, CD3^−^, CD5^−^, CD34^−^, CD68^−^, and MPO-), and a pleural effusion immunophenotyping (IFT) revealed involvement by large B-cell lymphoma. The diagnosis of mPBL/SBL DLBCL, germinal center phenotype, stage IV Ann Arbor and IVE IELSG, IPI score 5, was established. The patient was proposed to undergo immunochemotherapy (R-CHOP protocol) and bisphosphonates (zoledronic acid), but due to clinical and analytical worsening after the first cycle (ECOG status [PS > 2], *CTCAE* grade 3-4 liver, and hematological toxicities), the treatment was suspended and the patient was referred to the palliative unit for best supportive care. At present, she recovered from toxicities and remains with a good symptomatic control.

Criteria to define PBL is still in debate, and the last version of the *World Health Organization* (WHO) classification does not provide a clearly diagnostic definition of uPBL and mPBL.^5^ Patients with PBL are characterized generally by a localized bone pain. Our patient presented with a generalized bone pain, documented as multiple lytic lesions in PET/CT scan. In such cases, the differential diagnosis for lytic lesions is broad, such as multiple myeloma, osteoblastoma, primary/secondary bone lymphoma, eosinophilic granuloma, infection (osteomyelitis), hyperparathyroidism, metastasis (sarcoma, neuroblastoma), and Ewing sarcoma, among others.^17^ A bone lymphoma diagnosis is performed by histopathological examination, immunohistochemical staining, and molecular analyses,^18^ and PET-CT is a standard tool for staging and response assessment for FDG-avid lymphomas, although this has not been established in PBL.^19^

The clinical presentation in our case might suggest a plasma cell disorder due to the presence of one of CRAB criteria (bone lytic lesions), but the absence of monoclonal plasma cells in bone marrow aspirate ruled out MM. Bone DLBCL diagnosis was confirmed by biopsy, and a PET-CT scan revealed an extensive hypermetabolic bone malignancy involvement and lower metabolism at the left cervical lymph node and right lower lobe. Pleural effusion immunophenotyping revealed involvement by large B-cell lymphoma, raising the following question: Are we facing SBL with an extensive bone involvement or mPBL with lymph node and lung involvement? Our opinion is that, although it has been documented an extranodal (lung) by IFT and lymph node (not proven by biopsy) involvement, due to the discrepancy of extensive bone and nodal involvement, we classify this case as mPBL. The literature is scarce about this issue. Huanwen Wu et colleagues^2^ evaluated patient characteristics and survival and prognostic factors in uPBL, mPBL, and SBL and showed that patients with mPBL and SBL share similar clinical (higher frequency of B symptoms, lymph node, and bone marrow involvement) and demographic characteristics and treatment outcomes compared with those with uPBL, suggesting that patients with mPBL should be better classified and treated as SBL.^2^

This case illustrates a rare presentation of DLBCL as bone lymphoma and raises the question as mentioned before: classify this case as mPBL or SBL? In mPBL, especially in those with regional lymph node and/or adjacent soft-tissue involvement, it may be impossible clinically and radiologically to distinguish from SBL, and this issue remains controversial throughout the literature. Better clarification of the diagnostic criteria and prospective studies are necessary to distinguish these two entities.
